# Bio-Interface on Freestanding Nanosheet of Microelectromechanical System Optical Interferometric Immunosensor for Label-Free Attomolar Prostate Cancer Marker Detection

**DOI:** 10.3390/s22041356

**Published:** 2022-02-10

**Authors:** Tomoya Maeda, Ryoto Kanamori, Yong-Joon Choi, Miki Taki, Toshihiko Noda, Kazuaki Sawada, Kazuhiro Takahashi

**Affiliations:** 1 Department of Electrical and Electronic Information Engineering, Toyohashi University of Technology, Toyohashi 441-8580, Japan; maeda.tomoya.rk@tut.jp (T.M.); kanamori.ryoto.kh@tut.jp (R.K.); choi@ee.tut.ac.jp (Y.-J.C.); taki.miki.wz@tut.jp (M.T.); noda.toshihiko.zk@tut.jp (T.N.); kazuaki.sawada@tut.jp (K.S.); 2 Electronics Inspired-Interdisciplinary Research Institute (EIIRIS), Toyohashi University of Technology, Toyohashi 441-8580, Japan

**Keywords:** microelectromechanical systems, immunosensor, Fabry–Pérot interferometer, surface-stress sensor, biosolid interface, prostate-specific antigen

## Abstract

Various biosensors that are based on microfabrication technology have been developed as point-of-care testing devices for disease screening. The Fabry–Pérot interferometric (FPI) surface-stress sensor was developed to improve detection sensitivity by performing label-free biomarker detection as a nanomechanical deflection of a freestanding membrane to adsorb the molecules. However, chemically functionalizing the freestanding nanosheet with excellent stress sensitivity for selective molecular detection may cause the surface chemical reaction to deteriorate the nanosheet quality. In this study, we developed a minimally invasive chemical functionalization technique to create a biosolid interface on the freestanding nanosheet of a microelectromechanical system optical interferometric surface-stress immunosensor. For receptor immobilization, glutaraldehyde cross-linking on the surface of the amino-functionalized parylene membrane reduced the shape variation of the freestanding nanosheet to 1/5–1/10 of the previous study and achieved a yield of 95%. In addition, the FPI surface-stress sensor demonstrated molecular selectivity and concentration dependence for prostate-specific antigen with a dynamic range of concentrations from 100 ag/mL to 1 µg/mL. In addition, the minimum limit of detection of the proposed sensor was 2,000,000 times lower than that of the conventional nanomechanical cantilevers.

## 1. Introduction

Cancer is one of the leading causes of premature death (age 30–69 years) in 134 out of 183 countries [1], with the number of deaths increasing by 9.3 million from 2000 to 2019 [2]. Therefore, screening methods for the early detection of cancer and other diseases are being actively studied. One such method is to measure biomarkers which are biomolecules in the blood, such as proteins, whose concentrations change according to the presence or progression of the disease [3]. They are primarily used as surrogate endpoints for therapeutic intervention trials in the medical field [4,5,6]. Prostate-specific antigen (PSA), the target of this study, is a biomarker that specifically changes its concentration in prostate cancer; blood levels with more than 4 ng/mL PSA concentration increase the risk of prostate cancer [7,8]. Enzyme-linked immunosorbent assay (ELISA) [9] is a widely used diagnostic analytical technique. For high-sensitivity detection using ELISA, isolating each biomolecule-enzyme complex into ultra-small reactor volume components is an effective strategy. Digital ELISA is a novel device that utilizes arrays of femtoliter chambers to realize the high-sensitivity detection of protein biomarkers that are labeled magnetic beads for digital counting; its limit of detection (LOD) is 60 ag/mL for PSA measurements [10]. However, it is a time-consuming process and requires skilled personnel for fluorescent labeling. In addition, they are large and expensive, making them unsuitable for on-site diagnostics and point-of-care testing (POCT) [11]. Therefore, label-free biosensors using miniaturized sensor elements and semiconductor microfabrication technology have been investigated as alternatives to ELISA.

Biosensors that are based on field-effect transistors (FET), categorized as electrochemical biosensors, are one of the most common and major types of biosensors that are suitable for POCT. They are capable of real-time electrical measurement and are expected to be miniaturized and arrayed by applying complementary metal oxide semiconductor (CMOS) technology [12]. However, FET-based biosensors face difficulty in measuring macromolecules such as proteins in high ionic strength solutions that are close to the physiological environment due to their Debye length limitation [13,14,15]. The Debye length is the distance at which 63% protein charges are screened out in charge screening effect. It is approximately 0.7 nm for FET channel in physiological salt environment, which is much smaller than the size of a regular IgG antibody (5~10 nm) [15]. Therefore, it is necessary to dilute the analyte by 10–10,000 fold for reducing the ionic strength [16,17]. However, this causes degradation of minimum LOD concentration and the loss of protein activity, stability, and binding affinity [18,19].

As Debye-shielding-free sensors, biosensors that are based on microelectromechanical systems (MEMS) can capture the mechanical response to the adsorption of molecules. They can be tailored to distinctive forms and dimensions and be designed to combine large numbers of elements [20]. As a result, they have been a source of great inspiration for various research findings in the field of biosensing applications. Microcantilever (MCL) biosensors have been reported [21] to detect PSA, and most of them can detect biological responses by (a) the dynamic mode that measures the resonance frequency change that is caused by the mass change [22,23], or (b) the static mode that measures the deflection that is caused by the change in surface stress [24,25]. The former can measure the amount of adsorbed molecules with high sensitivity and accuracy; however, its vibration quality is highly dampened in aqueous media [26]. In contrast, although the latter is suitable for real-time measurements in liquids and the establishment of multidimensional arrayed sensors, the surface-stress sensor was inferior in the minimum LOD by two to three orders of magnitude.

To resolve these shortcomings, we previously proposed a surface-stress sensor using a Fabry–Pérot interferometer (FPI) to improve the signal conversion efficiency of the surface stress. It demonstrated on-chip molecular detection by integrating with photodetectors and CMOS circuitry that was based on the CMOS image sensor technology for POCT applications [27,28]. In contrast to conventional surface-stress sensors with a silicon (Si) piezoresistive cantilever, this optical interferometric surface-stress sensor employs a low Young’s modulus freestanding membrane for high stress sensitivity; in particular, it utilizes a nanosheet transfer technique that forms a freestanding elastomer membrane and can significantly improve the surface-stress sensitivity [29] along with successful detection of 1 fg/mL human serum albumin (HSA) antigen under physiological conditions [30]. However, owing to the surface chemical treatment process during the biosolid interface formation, the nanometer-thick deformable membrane mechanically degrades, causing a variation in its membrane shape before the antigen-antibody reaction. Unlike conventional FET biosensors with a sensing area that is fixed to a substrate and MEMS-based biosensors such as MCL with high-stiffness deformable parts, minimally invasive chemical processing can provide freestanding and soft nanosheets for the optical interferometric sensor. In this study, we propose a minimally invasive chemical functionalization method to form a biosolid interface that reduces deformable membrane degradation and a resultant air gap variation while maintaining a high sensitivity of the freestanding membrane. In addition, the improved sensor demonstrates label-free attomolar detection of PSA.

## 2. Materials and Methods

### 2.1. Structure and Operation of MEMS Optical Interferometric Surface-Stress Immunosensor

**Figure 1:**
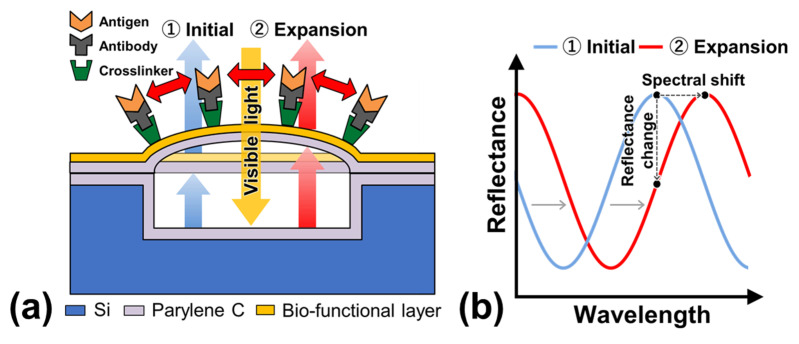
(**a**) Cross-sectional schematic diagram of a MEMS optical interferometric surface-stress immunosensor. “Si” represents silicon. (**b**) Schematic image of the reflection spectrum with interference peaks that were determined by an airgap. The peak positions shift depending on the nanomechanical deflection that is associated with molecular adsorption.

Figure 1a displays a schematic diagram of the cross-sectional structure and detection principle of the optical interferometric surface-stress sensor. The proposed sensor was composed of a parylene-C nanosheet with a bio-functional layer to represent the deformable membrane on a cavity that was formed on a Si substrate; therefore, the FPI was composed of a deformable membrane, air gap, and Si substrate. The antigen-antibody binding deformed the membrane due to repulsive forces between the charges of the bound antigen. When the deformable membrane was deflected by the surface stress, the distance between the Si substrate and the deformed membrane increased, thereby causing a spectral shift to the longer wavelength (Figure 1b). Therefore, the nanomechanical deflection of the deformable membrane could be evaluated by observing the reflection spectral shifts. Since the deflection amount of the surface-stress sensor is inversely proportional to the Young’s modulus of the material of the moving part and the thickness of the film, the detection sensitivity can be improved using a soft material with a low Young’s modulus and a thin film [29,30]. In this study, parylene-C was utilized as a support layer of the freestanding membrane since its Young’s modulus is two orders of magnitude lower than that of Si, and its uniform nanosheets of submicron thickness can be easily obtained by chemical vapor deposition at room temperature (25 °C) [31].

### 2.2. Cavity Formation by Nanosheet Transfer

**Figure 2:**
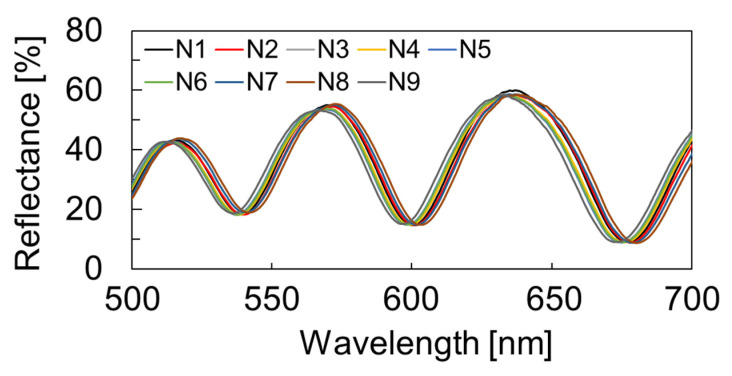
The measured reflection spectra of the FPI sensors that were fabricated by nanosheet transfer process. A sensor array with a uniform airgap with a variation of up to 33 nm was formed. N1-9 represent the nine measured FPI sensor elements.

The sealed cavity structure was developed using the proposed parylene-C transfer process [30] (Figure 2); it is a dry transfer technique that uses thermal bonding between parylene-C layers, thereby enabling the fabrication of an MEMS-based surface-stress immunosensor with a submicron-thick freestanding membrane on a 300 µm diameter cavity that maintains air inside the cavity during the wet process.

In optical interferometric sensors, the amount of spectrum shift that is associated with membrane deflection depends on the initial air gap [32]. Therefore, if the initial air gap varies from cavity to cavity, the signal conversion efficiency for membrane deflection at the gap expansion will be different for each sensor. In a previous study [30], FPI sensors that were as close as possible to the standard optical interference spectrum were selected for immunosensing. However, considering future multi-cavity measurements by arraying, reducing this variability is crucial to obtain a more accurate measurement. The tolerance of the variation can be discussed using the free spectral range (FSR) of the interference property. FSR ($\Delta \lambda_{\mathrm{FSR}}$) is the spacing in the optical wavelength between two successive reflected or transmitted optical intensity maxima or minima of an interferometer. It is calculated using the following equation:

(1) $$\Delta \lambda_{\mathrm{FSR}}\approx \frac{\lambda^{2}}{2nd}$$

where *λ*, n, and d represents the wavelength of the interference peak, refractive index of air, and air gap length, respectively. Conventionally, we measure peaks near a specific wavelength (around 550–600 nm); however, a spectral deviation outside the FSR due to the air gap variation can cause incorrect measurement of the different interference orders.

Since the spectral shift is inversely proportional to the interference order *m* [32], it is necessary to evaluate peaks of the same order. The allowable gap variation (∆*d*) for the peaks to stay within the FSR was calculated using the following equation:

(2) $$\Delta d=\frac{m\cdot \Delta \lambda_{\mathrm{FSR}}}{4n}$$

The reflection spectra of the sensors arrayed in the chip were evaluated using microspectroscopy. As displayed in Figure 2, the variation was less than 33 nm, indicating that the proposed nanosheet transfer technique allowed a uniform cavity under the deformable membrane.

### 2.3. Molecular Modification Using Oxidized Poly Methyl Methacrylate (PMMA)

In our previous study [30], we measured the reflection spectrum of an anti-HSA modified PMMA layer that was treated with an HSA antibody on a deformable membrane (Figure S1a), and successfully detected 1 fg/mL HSA antigen. To modify the surface of the deformable membrane with biomolecules, the surface of the PMMA was oxidized to form carboxyl groups using UV/O_3_ treatment [33]. The UV/O_3_ treatment was carried out at room temperature at 25 °C with the flow rate of O_2_ at 0.5 L/min. For crosslinking between the antibody and the membrane surface, 1-ethyl-3-(3-dimethylaminopropyl) carbodiimide (EDC) 5 mg/N-hydroxysuccinimide (NHS) 1.5 mg in 1 mL of 2- (N-morpholino) ethane sulfonic acid (MES buffer) was used. It was performed for 30 min to bind the amino groups of the antibody. For antibody immobilization, the chip was dipped into a 10 μg/mL solution of anti-PSA antibody in phosphate-buffered saline (PBS) for 2 h. To prevent nonspecific adsorption, it was immersed in 100 μg/mL bovine serum albumin (BSA) for 2 h as a blocking agent.

Although the measurements were performed using sensors on the same chip, an air gap variation (membrane shape variation) was observed for each sensor due to the surface chemical treatment for antibody immobilization, including blocking. FPI sensors with different membrane shapes before immunosensing are considered to have different sensitivities. In the abovementioned previous research, the sensor elements with the highest possible identical interference characteristics were selected. However, as the number of sensors increases, it is difficult to select them beforehand using spectroscopic measurement to perform multiple measurements.

### 2.4. Molecular Modification Using Amino-Functionalized Membrane

To obtain antibody modification to the freestanding parylene-C nanosheet without UV irradiation, we investigated the use of glutaraldehyde (GA) as the cross-linker. GA has an aldehyde group (CHO) at both ends of the molecular sequence that allows cross-linking between amino groups without UV irradiation, and the reaction is almost irreversible at pH 7–9 [34]. Therefore, it firmly anchors proteins such as enzymes and antibodies to the amino-functionalized surface and has, as a result, found the widest application in various fields [35].

**Figure 3:**
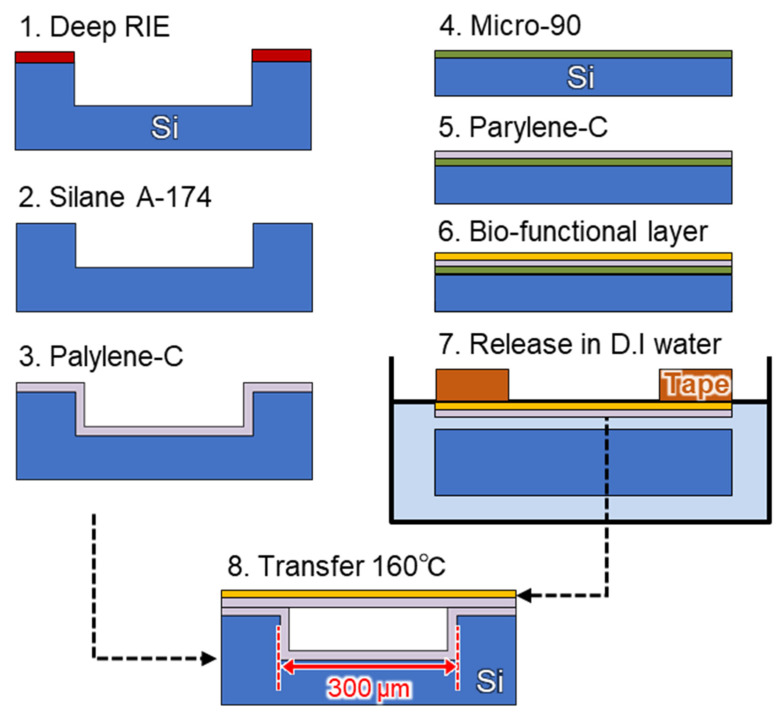
Fabrication procedure for the microelectromechanical system (MEMS) optical interferometric surface-stress immunosensor. (1) Preparing the silicon substrate with micro-cavity structure by deep reactive ion etching (RIE). (2) Silane-coupling treatment using a mixture of deionized water (DIW), isopropyl alcohol (IPA), and γ-methacryloxypropyltrimethoxysilane (A-174) in a ratio of 100:100:1. (3) Deposition of parylene-C for a binder layer using a parylene deposition system. (4) Spin-coating surfactant (Micro-90 2%) on a two-inch silicon substrate. (5) Deposition of parylene-C for a transfer sheet. (6) Formation of a bio-functional layer on the parylene-C layer. (7) The parylene-C nanosheet with the bio-functional layer supported by Kapton tape was released from the silicon substrate in DIW. (8) The released nanosheet was transferred onto the silicon substrate with binder parylene-C and heated at 160 °C for 1 h on a hot plate. Finally, it was cut into 2.5 mm × 2.5 mm sizes using a laser dicing machine.

**Figure 4:**
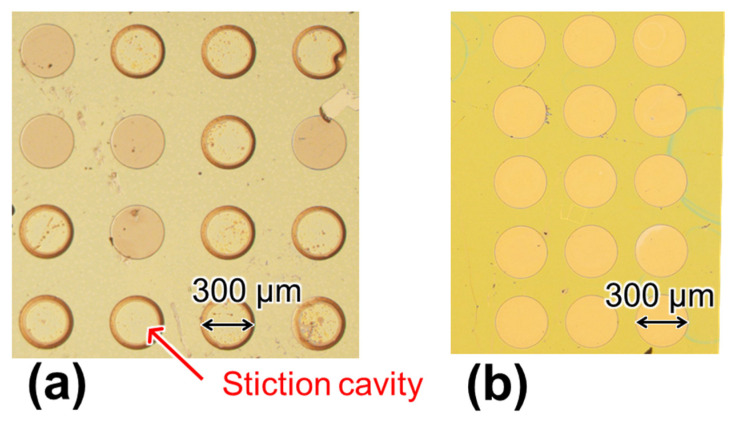
Chip photograph after molecular modification using (**a**) a PMMA film and UV irradiation and (**b**) an amino-functionalized parylene (diX-AM) layer and glutaraldehyde cross-linking. The defective rate of deformable membrane was decreased.

In this study, we used diX-AM (KISCO) as a bio-functional layer since it has the same basic structure as parylene-C along with an amino group at the end of the molecular sequence. The typical deposition thickness of diX-AM, that is obtained by using the chemical vapor deposition (CVD) process, is 10–20 nm, which is 1/8 thinner than that of PMMA. Because it is difficult to form a thick film, parylene-C is used as the support layer to maintain the freestanding membrane. diX-AM was deposited using a parylene deposition system (PDS 2010, Parylene Japan K. K., Tokyo, Japan) in three steps: (a) evaporation of the dimer at 135 °C, (b) pyrolysis to generate p-xylene radicals at 600 °C, and (c) deposition on the substrate at room temperature (25 °C) (Figure 3, step 6). diX-AM thickness was evaluated using a spectroscopic film thickness measurement system (VM-1230, Screen Semiconductor Solutions Co. Ltd., Kyoto, Japan). The surface modification protocol for selective detection of PSA is summarized in the Supplementary Materials in Figure S1b. GA cross-linking to amino-functionalized parylene has been reported in several studies [36,37]. In this study, the sensor chips were treated with 2.5% GA in PBS for 30 min. The uniform immobilization of antibody molecules using GA cross-linking was confirmed by fluorescence observation (Figure S2). PSA antibody modification and BSA blocking were performed as per the method that was discussed in Section 2.3. Since this method was expected to prevent the yield reduction and the air gap variation by the degradation of parylene-C, the design gap was changed from 4.0 to 2.4 µm (Figure 3, step 1). Although the air gap length was shortened, the FPI cavity yield was over 95%, owing to the low invasive molecular modification (Figure 4). According to equation (1), the narrower the gap, the larger the value of FSR, resulting in an increased tolerance to gap variation. For the spectral measurement that is described below, the cavities without wrinkles and particles were selected.

### 2.5. Zeta Potential Measurement

To confirm whether the target surface molecular state was achieved, the surface zeta potential was evaluated using the flow potential measurement method [38]. For evaluating the zeta potential, we prepared 10 mm × 10 mm substrates with the following surface conditions: pristine parylene-C, diX-AM-coated parylene-C layer, and a surface that was treated in the following order: GA, anti-PSA, and BSA. Each surface condition was measured using the zeta potential measurement system (ELSZ-2000, Otsuka Electronics, Osaka, Japan). A 10 mM NaCl solution was measured at pH 6.87 using a pH meter (LAQUA F-72, HORIBA Advanced Tech., Tokyo, Japan) for the measurement solution, and polystyrene latex particles (diameter 500 nm, Otsuka Electronics, Osaka, Japan) that were coated with hydroxypropyl cellulose were used for mobility measurements.

### 2.6. Label-Free PSA Detection Using Optical Interferometry

**Figure 5:**
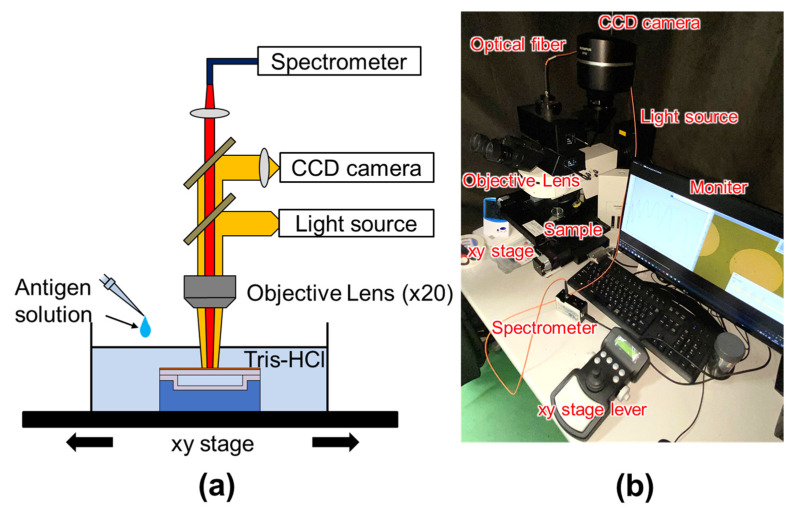
(**a**) Schematic and (**b**) photograph of the measurement setup for detecting membrane deformation in a surface-stress sensor. The deformable membrane was exposed by white light using a halogen lamp. Reflection spectrum that was generated by optical interference between the deformable membrane and the silicon substrate was acquired with a spectroscope (USB4000, Ocean Optics) through a 20X objective lens and an optical fiber with a core diameter of 400 µm, resulting in 20 µm spot diameter. Multi-measurement for reflection spectra of arrayed cavity was performed using a 2D motorized positioning stage (H105, PRIOR).

For sensitivity evaluation, the reflection spectral shift due to the antigen-antibody reaction was measured using microspectroscopy (Figure 5). The measurements were performed using sensors with a deformable membrane thickness of 115 nm. The PSA antigen was purchased from SCRUM Inc. (Tokyo, Japan) and provided as a powder. The affinity-purified anti-PSA antibody with a concentration of 1.26 mg/mL was also purchased from SCRUM Inc. The sensor chips were stabilized in a buffer solution of 1.9 mL Tris-HCl before the measurement.

## 3. Results and Discussion

### 3.1. Deformed Shape Evaluation of Molecular Modified Membrane

**Figure 6:**
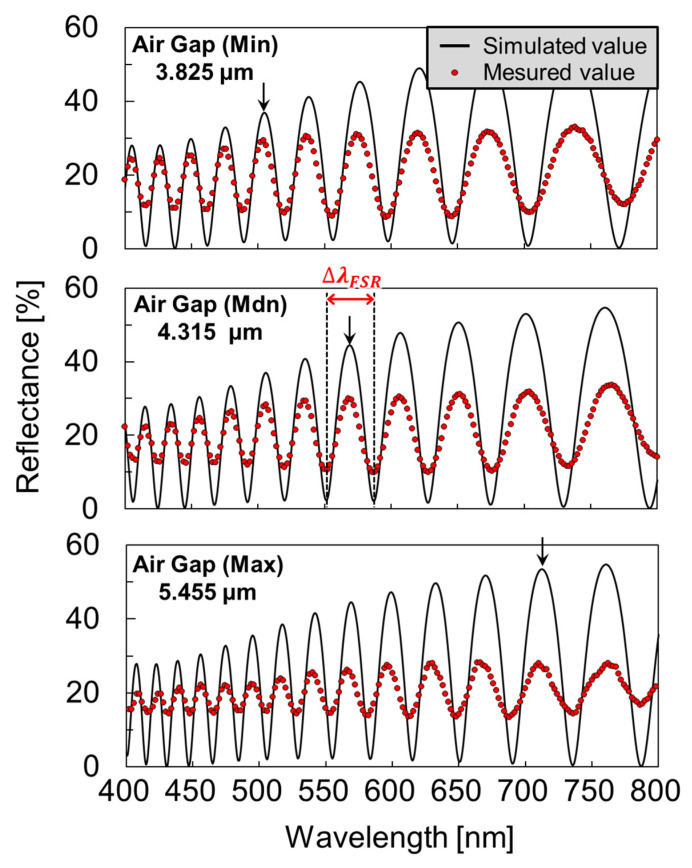
The reflection spectrum of the antibody-modified FPI sensors using an oxidized poly methyl methacrylate (PMMA) with fitting curves. The shape of the deformable membrane varies due to the surface chemical reaction. The minimum (Min), median (Mdn), and maximum (Max) airgaps are 3.825 µm, 4.315 µm, and 5.445 µm, respectively. Although the deviation of the light intensity can be attributed to surface scattering and deformed shape of membrane (upper half-mirror), it does not affect the airgap evaluation since interference peak positions are determined solely by the optical pass length (multiplication of gap and refractive index) between two half-mirrors.

Figure 6 illustrates the reflection spectra of the surface-modified FPI sensors with a PMMA layer corresponding to the maximum, median, and minimum air gaps of the measured nine reflection spectra (three cavities per chip, three chips in total) before dropping the antigen reagent. The solid line represents a fitting curve for calculating the air gap using an optical analysis software that was based on the rigorous coupled-wave analysis (RCWA) method (RSOFT DiffractMOD, Synopsys Inc., Mountain View, CA, USA). The parameters for the thickness of the parylene-C layers as a binder and a deformable membrane, and the PMMA layer were set to 100 nm, 100 nm, and 120 nm, respectively. The arrows indicate the peaks of the same interference order (*m* = 16). Focusing on the peak at the 586 nm wavelength in the interference spectrum with a median gap of 4.439 µm, from equation 2, the gap variation Δd within the FSR range was approximately 0.145 µm (Figure 6). The air gap varied from 3.825 µm to 5.455 µm, and only approximately 33% of the cavities were within the Δd range.

One possible reason for this air gap difference is the degradation of the parylene-C membranes due to UV irradiation for the PMMA oxidation that converts the end of the surface molecular structure to carboxyl groups (-COOH) [39]. Reports confirmed that the thermal stability and electrical properties of parylene-C are degraded by UV irradiation, causing embrittlement [40,41]. Depending on the UV irradiation time, up to 95% of the sensors in the chip were found to be defective; these defects included contact between the deformable membrane and the silicon substrate or distorted deformation [30]. As a result, the sensors did not work as optical interference immunosensors (Figure 4). In contrast, as mentioned in Section 2, the parylene-C membrane in the proposed sensor exhibited a high-affinity material. Therefore, in this study, we investigated an antibody modification method to suppress the variation in the air gap within the Δd range and improve the yield.

**Figure 7:**
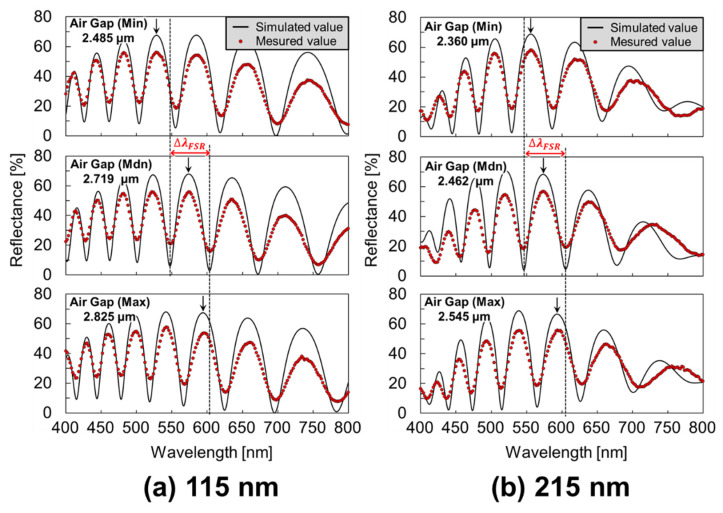
Reflection spectrum of the antibody-modified FPI sensors with a diX-AM/parylene-C membrane thickness of (**a**) 115 nm and (**b**) 215 nm. The arrows indicate the position of the same interference order at the minimum (Min), median (Mdn), and maximum (Max) airgaps for evaluation of variation.

Figure 7a,b display the difference in the reflection spectrum with a diX-AM layer corresponding to the air gap under both thickness conditions of the deformable membrane. To confirm the relationship between the deformable membrane thickness and the variation in the deformed film shape (i.e., variation in the air gap), FPI sensors with a parylene-C thickness of 100–200 nm were prepared. The maximum, median, and minimum air gaps are represented in the measurement of the reflection spectra of the nine sensors (three cavities per chip, three chips in total). For curve fitting, the analysis parameters for the thickness of parylene-C as a binder and a deformable membrane, and the diX-AM layer were set to 100 nm, 100 or 200 nm, and 15 nm, respectively. For FPI sensors with a total deformable membrane thickness of 115 nm, the median air gap after antibody modification, including BSA blocking, was 2.719 µm. Focusing on the 11th-order interference peak was obtained at 575 nm, the air gap tolerance ∆*d* was approximately 0.152 µm and 89% (8/9) of the cavities were within the ∆*d* range. For the samples with a total deformable membrane thickness of 215 nm, the median air gap was 2.462 µm, and the interference peak was observed at 574 nm was *m* = 10, owing to the narrow gap. All the sensors were measured within ∆*d* (approximately 0.153 µm), thereby confirming further improvement in shape variation. The results indicated that GA cross-linking on amino-functionalized membranes could be used to evaluate a peak of the same interference order in the same wavelength range.

**Figure 8:**
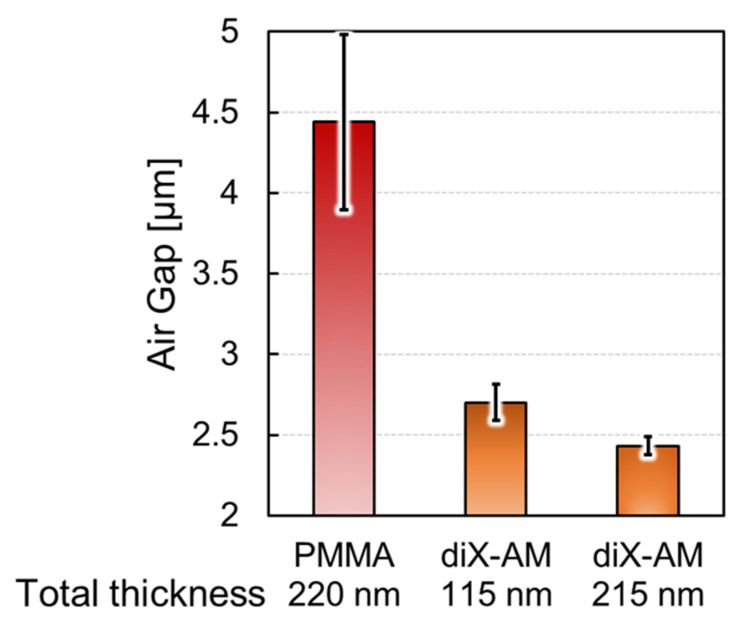
Comparison of the airgap variations that represent variations in the deformed membrane shape by applying the receptor modification method for oxidized poly methyl methacrylate (PMMA) and diX-AM. The airgap variation of the diX-AM coated sensors decreased to 1/5 to 1/10.

To compare the previous and proposed modification methods, we evaluated the variation of the deformed membrane that was associated with antibody immobilization, as displayed in Figure 8.

The error bars indicate the corresponding standard deviations. The standard deviation of the air gap length under the receptor-modified membrane was reduced to 1/5 and 1/10 of the previous FPI sensor at the deformed membrane thickness of 115 nm and 215 nm, respectively. This indicated that the proposed method successfully suppressed the membrane shape variation, and the stability could be further improved by changing the deformable membrane thickness parameter within the submicron range. It should be noted that surface-stress sensitivity and the variation of the deformed membrane shape have a trade-off relationship since the surface-stress sensitivity is inversely proportional to the thickness of the deformable membrane. However, we expected sufficient sensitivity since the variation that was suppressed in the film thickness was in the submicron range.

### 3.2. Surface Condition Evaluation by Zeta Potential Measurement

**Figure 9:**
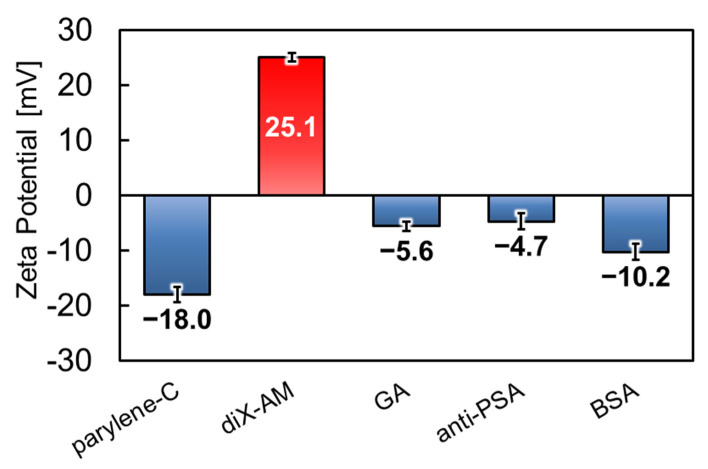
Zeta potential evaluation of five different surface conditions (parylene-C surface, diX-AM surface, and modified surfaces with GA, anti-prostate specific antigen (PSA), bovine serum albumin (BSA), respectively) at each stage with measurements conducted three times.

Figure 9 presents the results of the five surface conditions, namely parylene-C, diX-AM, GA, anti-PSA, and BSA, respectively, at each stage with three measurements. The error bars indicate the standard deviations. In the pristine parylene-C layer, the surface zeta potential was −18.3 mV, which is consistent with the values of other studies [42,43]. When diX-AM was deposited on parylene-C, the zeta potential reversed and increased to +25.1 mV. This was probably due to the contribution of the amino groups on the diX-AM surface. Since the zeta potential of the amino-functionalized particles has been found to yield 10–40 mV in the pH range of 6–7 [44,45], a positive change was considered appropriate. Since the polarity of the zeta potential was reversed, it is considered that the surface of parylene-C was covered with diX-AM with a thickness of 10–20 nm. After GA treatment, the measured value was −5.6 mV. Since GA has no dissociating group, the surface loses its polarity when the amino group of diX-AM is terminated [46,47]. Therefore, GA cross-linking was expected to decrease the zeta potential. When anti-PSA was cross-linked by GA, the zeta potential was −4.7 mV. Since anti-PSA, with an isoelectric point of around 5.5~6.0 [48,49], is expected to be negatively charged in a solution at pH 6.87, the result was appropriate. Finally, after the blocking treatment with the BSA solution, the zeta potential decreased to −10.2 mV. The isoelectric point of BSA is approximately 4.7~5.1 [50,51], which is lower than that of the anti-PSA. Therefore, when BSA adsorbs specifically to residues of GA or non-specifically to unterminated amino groups, the zeta potential is expected to move further in the negative direction. In summary, the zeta potential for each process is consistent with the intended molecular binding. In addition, the AFM images suggested that molecules such as antigens, antibodies, and cross-linking agents were adsorbed on the surface using the proposed modification protocol. (Figure S3) Therefore, the new protocol using diX-AM and GA cross-linking, which is less invasive than the previous study’s method using PMMA and EDC/NHS cross-linking, could also form a biosolid interface for selective adsorption of antigens.

### 3.3. Molecular Selectivity and Label-Free PSA Detection

**Figure 10:**
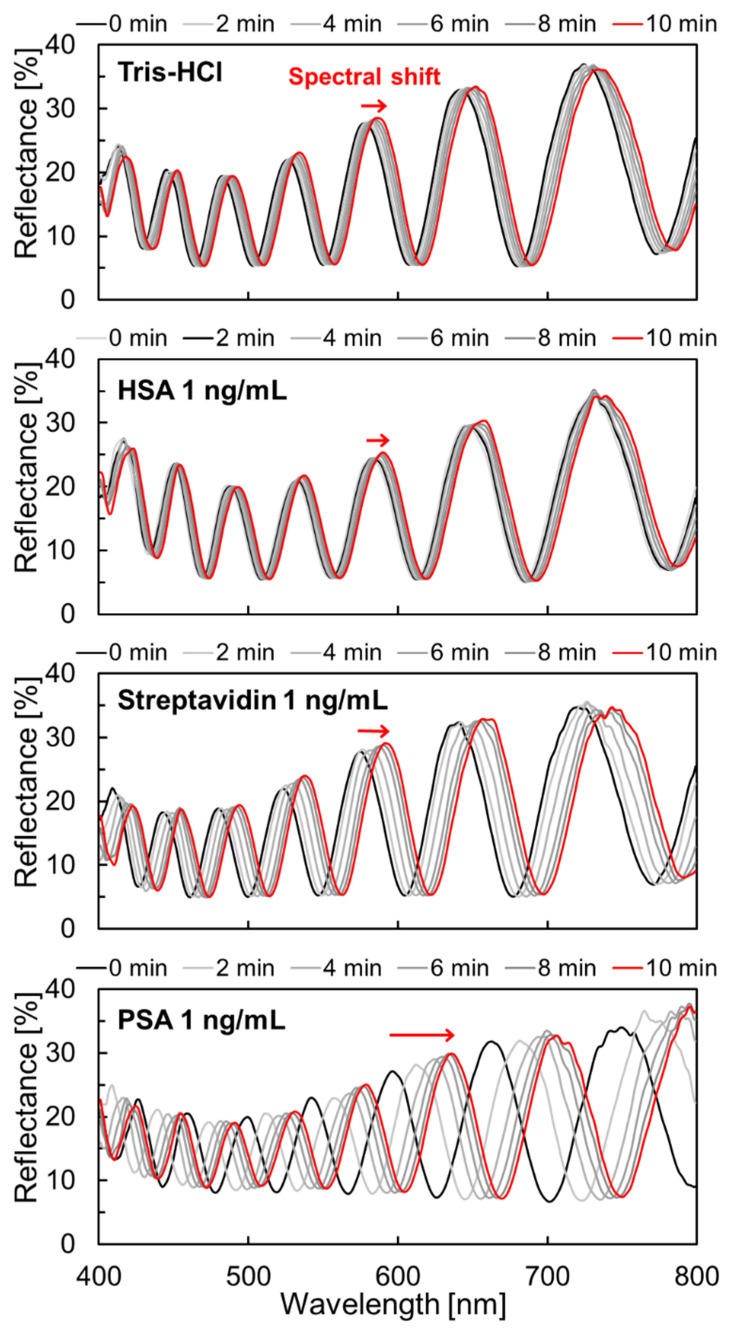
Result of the tracking the spectral shift after dropping reagent for Tris-HCl buffer (negative control) and 1 ng/mL solution (human serum albumin (HSA), Streptavidin, PSA).

The isoelectric point of PSA is approximately 6.9 [52,53], so proteins that were used for selectivity evaluation had to be the same or lower isoelectric point. Since the isoelectric points of HSA and Streptavidin are 5.3 [54] and 5~6 [55,56], respectively, a larger repulsive force compared with PSA is expected to be generated associated with adsorption on the surface. PSA antibody-modified sensors were reacted with dropping 0.1 mL Tris-HCl, HSA (Rockland Immunochemicals), Streptavidin (Sigma-Aldrich), and PSA in this measurement. All the protein solutions were adjusted to a final concentration of 1 ng/mL diluted by Tris-HCl using each protein in solution or powder form with guaranteed concentration or purity, and their corresponding spectral shifts were measured (Figure 10). When compared with the other solutions, the interference peak of the PSA-treated FPI sensor shifted to the long wavelength side after the drop.

**Figure 11:**
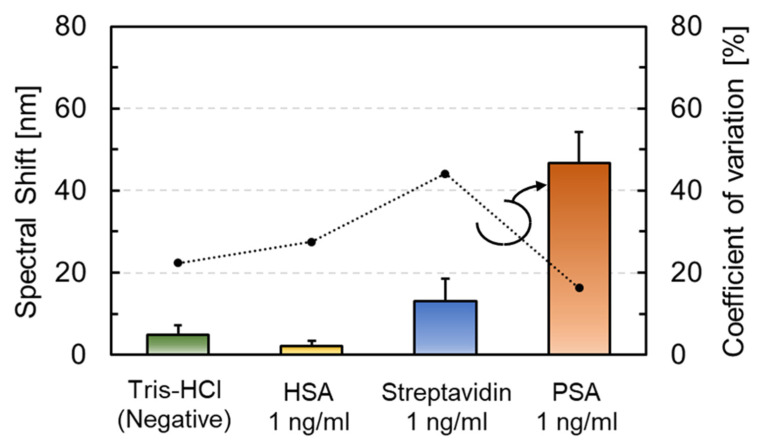
Molecular selectivity of anti-PSA-modified FPI sensor for Tris-HCl and 1 ng/mL solution (human serum albumin (HSA), Streptavidin, PSA).

Figure 11 displays the difference in the amount of spectral shift for dropping each solution. Each data point represents the average value (N = 9 or more), and the error bars indicate the corresponding standard deviation. The spectral shifts that were treated with the Tris-HCl buffer (negative control), HSA, Streptavidin, and PSA were 4.9 nm, 2.2 nm, 13 nm, and 46.6 nm, respectively. Although the amount of spectral shift of Streptavidin was larger than that of Tris-HCl as a negative control, it was only 1/4 of the response when compared with PSA, thereby confirming the difference between the specific and non-specific adsorption.

**Figure 12:**
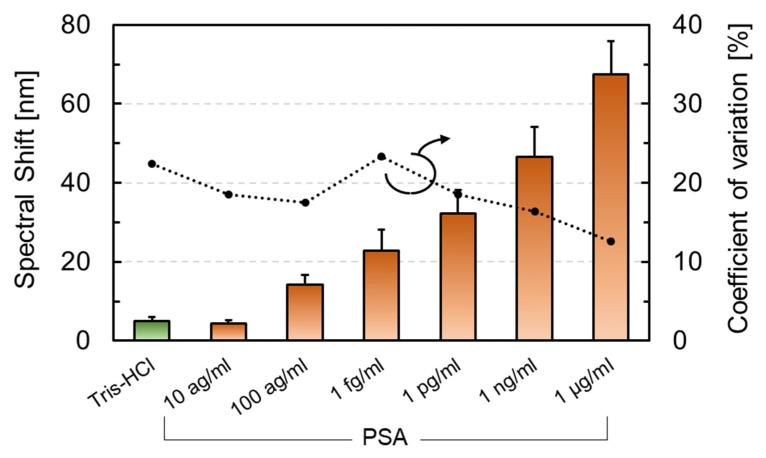
Spectral shift results after dropping buffer solution, including no PSA and PSA, at a final concentration of 10 ag/mL–1 µg/mL.

Figure 12 displays the spectral shift depending on the PSA concentration. The concentration of the PSA solutions was adjusted to final concentrations of 1 fg/mL, 1 pg/mL, 1 ng/mL, and 1 µg/mL. Thereafter, 0.1 mL solutions were added dropwise to the buffer solution in which the sensor chip was immersed. The average spectral shifts (N = 9 or more) that were treated with the buffer solution and the PSA solution at a concentration of 10^−17^, 10^−16^, 10^−15^, 10^−12^, 10^−9^, and 10^−6^ g/mL were 4.9 nm, 4.4 nm, 14.1 nm, 22.9 nm, 32.2 nm, 46.6 nm, and 67.4 nm, respectively. The amount of spectral shift increased with antigen concentration. Furthermore, there was a significant difference at the 100 ag/mL concentration compared to the negative control using Tris-HCl. This result was 2,000,000 times lower than that of the static-type microcantilever, and it exhibited the highest sensitivity for PSA detection among the label-free biosensors that were based on semiconductor microfabrication technology (Table 1).

## 4. Conclusions

In this study, we proposed a minimally invasive molecular modification technique for a MEMS optical interferometric surface-stress immunosensor to reduce deformable membrane degradation and variation of the deformed membrane shape. Depending on the deformable membrane thickness, by utilizing diX-AM and GA crosslinking without UV irradiation, the air gap variation under the antibody-modified suspended parylene-C nanosheet was successfully reduced to approximately 1/5–1/10 of the previous study, thereby providing a more accurate measurement for future multicavity measurements by arraying. The FPI sensors with improved chemical functional membranes demonstrated excellent molecular selectivity and an LOD of 100 ag/mL, which is comparable to 60 ag/mL of digital ELISA [10]. The nanosheet fabrication technique and optical interferometric transducer that were reported in this study were compatible with CMOS-MEMS integrated devices and did not require an external measurement system. Using the proposed techniques, a robust sensing system that compensates for false positive reactions due to physical responses or non-specific adsorption by differential amplification circuits can be built. We believe that it will enable a biosensor with high sensitivity and stability while providing rapid measurement and portability that is suitable for POCT devices.

## Figures and Tables

**Table 1:** Comparison of PSA biosensors using microfabrication technology for POCT.

Device Type	Operating Environment	LOD for PSA	Ref.
FET	in liquid (low ionic strength)	1 fg/mL	[57]
Microcantilever (Dynamic method)	in liquid	10 ng/mL	[22]
	air (25 °C)	1 pg/mL	[23]
Microcantilever (Static method)	in liquid	0.2 ng/mL	[24]
	in liquid	0.2 ng/mL	[25]
This study	in liquid	100 ag/mL	N/A

## Data Availability

The data presented in this study are available in supplementary material.

## Supplementary Materials

The following supporting information can be downloaded at: https://www.mdpi.com/article/10.3390/s22041356/s1. Figure S1. Comparison of Molecular modification protocol by using (a) oxidized poly methyl meth-acrylate (PMMA) and (b) diX-AM. Figure S2. Fluorescence image of FITC-conjugated BSA antigen (FITC-BSA) immobilized surface. The experiment was performed on a 10 mm × 10 mm diX-AM/parylene-C/Si substrate modified with GA. FITC-BSA solution with a concentration of 100 μg/mL was applied with drop wise to cover about half of the area of the substrate, and fluorescence observation was performed in the same substrate. Fluorescence images in the (a) border region, (b) FITC-BSA treatment area, and (c) FITC-BSA untreated area were obtained by 50X objective lens at the same exposure time. Figure S3. AFM images (1 × 1 μm) of (a) the surface of as-deposited diX-AM (RMS: 1.42 nm, P–V: 11.6 nm) and (b) the surface after antigen-antibody reaction at a PSA concentration of 1 μg/mL (RMS: 4.51 nm, P–V: 51.9 nm).
