# Supplementary material for: Bio-Interface on Freestanding Nanosheet of Microelectromechanical System Optical Interferometric Immunosensor for Label-Free Attomolar Prostate Cancer Marker Detection
Source: Sensors (Basel). 2022 Feb 10;22(4):1356. doi: 10.3390/s22041356 (PMC8963056; doi:10.3390/s22041356)
Supplement: Supplementary file 1 [file sensors-22-01356-s001.zip › sensors-1568540-supplementary.pdf]

sensors

Supplementary Materials

Bio-Interface on Freestanding Nanosheet of Microelectromechanical System Optical Interferometric Immunosensor for Label-Free Attomolar Prostate Cancer Marker Detection

Tomoya Maeda [1], Ryoto Kanamori [1], Yong-Joon Choi [1], Miki Taki [1], Toshihiko Noda [1,2], Kazuaki Sawada [1,2] and Kazuhiro Takahashi [1,*]

[1] Department of Electrical and Electronic Information Engineering, Toyohashi University of Technology, Toyohashi 441-8580, Japan; maeda.tomoya.rk@tut.jp (T.M.); kanamori.ryoto.kh@tut.jp (R.K.); choi@ee.tut.ac.jp (Y.-J.C.); taki.miki.wz@tut.jp (M.T.); noda.toshihiko.zk@tut.jp (T.N.); kazuaki.sawada@tut.jp (K.S.)

[2] Electronics Inspired-Interdisciplinary Research Institute (EIIRIS), Toyohashi University of Technology, Toyohashi 441-8580,-Japan

* Correspondence: takahashi@ee.tut.ac.jp; Tel.: +81-532-44-6740

Citation: Maeda, T.; Kanamori, R.; Choi, Y.-J.; Taki, M.; Noda, T.; Sawada, K. Bio-Interface on Freestanding Nanosheet of Microelectromechanical System Optical Interferometric Immunosensor for Label-Free Attomolar Prostate Cancer Marker Detection. *Sensors* **2022**, *22*, 1356. https://doi.org/10.3390/s22041356

Academic Editors: Wamadeva Balachandran and Zoltán Fekete

Received: 7 January 2022
Accepted: 6 February 2022
Published: 10 February 2022

Publisher's Note: MDPI stays neutral with regard to jurisdictional claims in published maps and institutional affiliations.

Copyright: © 2022 by the authors. Submitted for possible open access publication under the terms and conditions of the Creative Commons Attribution (CC BY) license (http://creativecommons.org/licenses/by/4.0/).

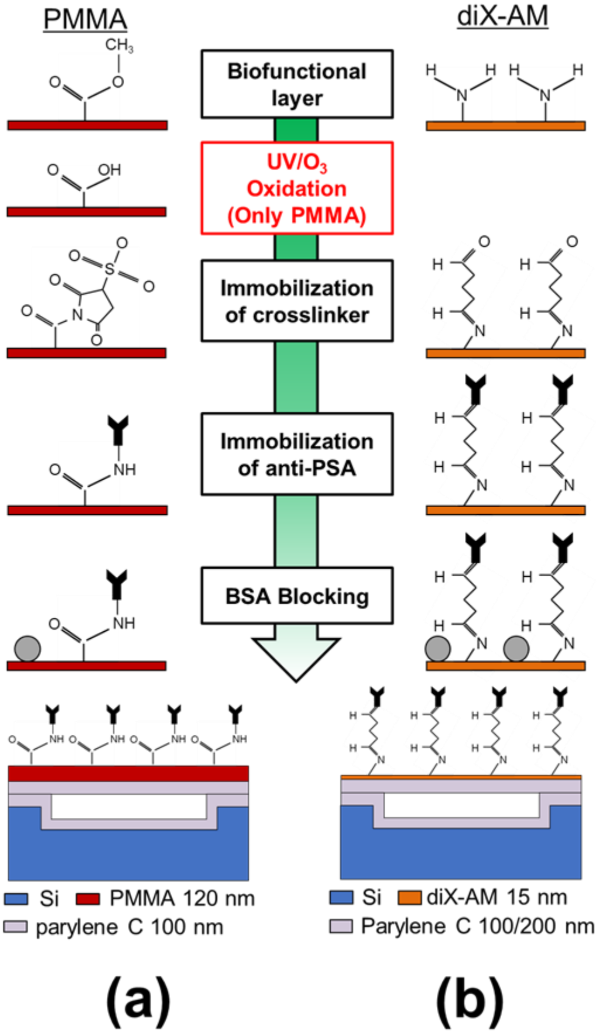

Figure S1. Comparison of Molecular modification protocol by using (**a**) oxidized poly methyl meth-acrylate (PMMA) and (**b**) diX-AM.

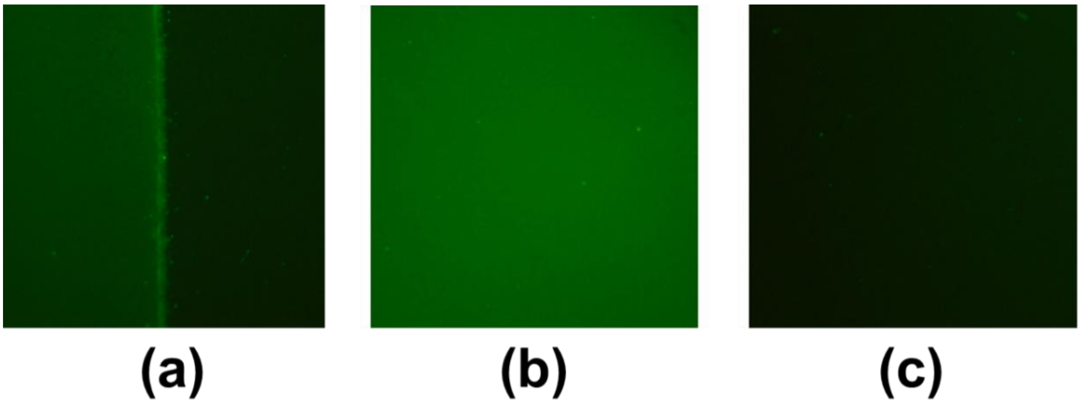

Figure S2. Fluorescence image of FITC-conjugated BSA antigen (FITC-BSA) immobilized surface. The experiment was performed on a 10 mm × 10 mm diX-AM/parylene-C/Si substrate modified with GA. FITC-BSA solution with a concentration of 100 μg/mL was applied with drop wise to cover about half of the area of the substrate, and fluorescence observation was performed in the same substrate. Fluorescence images in the (**a**) border region, (**b**) FITC-BSA treatment area, and (**c**) FITC-BSA untreated area were obtained by 50X objective lens at the same exposure time.

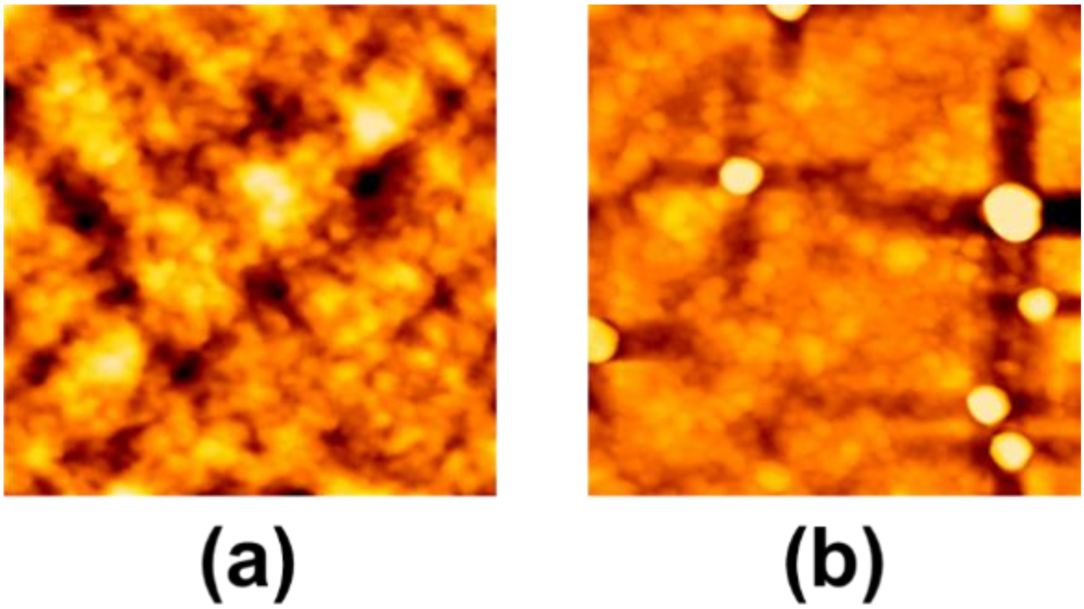

Figure S3. AFM images (1 × 1 μm) of (**a**) the surface of as-deposited diX-AM (RMS: 1.42 nm, P–V: 11.6 nm) and (**b**) the surface after antigen-antibody reaction at a PSA concentration of 1 μg/mL (RMS: 4.51 nm, P–V: 51.9 nm).
